# Hydroxytyrosol confers resilience against the depressive, anxiogenic and cognition-disruptive effects of chronic stress

**DOI:** 10.1016/j.ynstr.2026.100824

**Published:** 2026-05-22

**Authors:** Ariyawan Tantipongpiradet, Reeta Davis, Enrico A. Altieri, Kevin E. O'Connor, Keith J. Murphy

**Affiliations:** a Neurotherapeutics Research Group, UCD School of Biomolecular and Biomedical Science, UCD Conway Institute, University College Dublin, Belfield, Dublin 4, D04 V1W8, Ireland; b BiOrbic Bioeconomy Research Ireland Centre, UCD School of Biomolecular and Biomedical Science, UCD Conway Institute, University College Dublin, Belfield, Dublin 4, D04 V1W8, Ireland; c Nova Mentis Limited, Nova UCD, Belfield Innovation Park, University College Dublin, Belfield, Dublin, D04 F438, Ireland

**Keywords:** Memory consolidation, Learning, Neuronal plasticity

## Abstract

Our understanding of the causal mechanisms of disorders such as anxiety and depression remains rudimentary. There is a pressing need to develop disease-modifying and preventative therapies that can be administered early and reduce symptom emergence. Here, we used a novel chronic unpredictable restraint stress (CURS) model in female and male rats to investigate the protective potential of biotechnologically-produced hydroxytyrosol. The CURS model produced indicators of anxiety, depression, cognitive deficits and social dysfunction in the elevated plus maze, sucrose preference test, novel object recognition and social interaction, respectively. Oral dosing with hydroxytyrosol (50 mg/kg/day, oral jelly formulation) prior to and during the period of restraint stress successfully protected against the anxiety, mood, social and cognitive symptoms mediated by chronic stress. The effect of hydroxytyrosol on behaviour was accompanied by the prevention of stress-induced declines in dopamine and serotonin and increased serum levels of corticosterone. Using single-cell RNA sequencing (scRNA-seq) of the hippocampus, we identified transcriptional signatures associated with chronic stress and their normalisation by hydroxytyrosol. Chronic unpredictable restraint stress caused widespread transcriptional dysregulation across neurons, astrocytes, and microglia. Gene Ontology and KEGG analyses revealed stress-related dysregulation of glutamatergic, GABAergic, dopaminergic, and cholinergic transmission. Neurodegenerative disease-associated transcriptional modules were enriched in the genes dysregulated by chronic stress and pathways related to synaptic vesicle cycling, neurotransmitter release, oxidative phosphorylation, and protein translation were prominently disrupted. Hydroxytyrosol treatment markedly attenuated these transcriptional changes, preserving the expression of genes involved in synaptic signalling, mitochondrial integrity, and protein homeostasis, thereby protecting cognitive and mood/stress regulation functions. These findings demonstrate that hydroxytyrosol exerts broad neuroprotective and stress-resilience effects by preserving neuronal transcriptional homeostasis, identifying hydroxytyrosol as a potent dietary bioactive that can buffer the molecular and behavioural sequelae of chronic stress.

## 1 Introduction

The global burden of mental illness is a growing concern. Depression and anxiety disorders are among the leading causes of disability worldwide, affecting more than 300 million people (WHO, 2021). Depression is characterised by persistent sadness, diminished interest in activities, and a range of cognitive and somatic symptoms such as fatigue, impaired concentration, and changes in appetite or sleep (American Psychiatric Association, 2022; Fried and Nesse, 2015). Anxiety disorders are marked by excessive worry, hyperarousal, and somatic symptoms like restlessness and include a range of conditions, such as generalised anxiety disorder, social anxiety disorder, and panic disorder (Bandelow and Michaelis, 2015). Both conditions show low and variable response rates to current treatments with notable side effects and high relapse potential (Rush et al., 2006, Stein Murray and Sareen, Lader, 2011). There is an urgent need for safer, more effective, and accessible treatment strategies that preserve mental health, particularly cognitive functions.

Cognitive decline is a hallmark of ageing and a key feature of many neurodegenerative and neuropsychiatric conditions, like depression and anxiety (Harada et al., 2013). It is increasingly clear that there is a bidirectional relationship in which emotional dysregulation worsens cognitive impairments, and vice versa (Deary et al., 2009; Petersen et al., 2018). Cognitive dysfunction can reduce coping capacity and resilience, heightening vulnerability to psychosocial stressors and worsening psychiatric conditions. Specifically, social cognition, including empathy, emotion recognition, and theory of mind, is also compromised in various mental illnesses (Adolphs, 2009). Impaired social cognition increases the risk of social withdrawal, isolation and loneliness, strong predictors of depression and anxiety (Cotter et al., 2018).

Hydroxytyrosol (HT; 4-(2-Hydroxyethyl)benzene-1,2-diol), a phenylethanoid compound found in olives, olive oil and other plant species, has emerged as a promising candidate for mental health neuroprotection. Known for its potent antioxidant, anti-inflammatory, and neuroprotective properties, HT is one of the key bioactive substances associated with the health benefits of the Mediterranean diet, which is well established to decrease risk of neuropsychiatric and neurodegenerative disease (Zhao et al., 2021; Fan et al., 2023; Chen et al., 2021; Perez-Barron et al., 2021a). Although hydroxytyrosol is most strongly associated with olive-derived products, it is also present in a range of plants, for example *Buddleja cordata* and *Cistanche tubulosa*, extracts of which are used in traditional medicine in Asia and America and have been shown to possess neuroprotective and antidepressant-like properties (Fan et al., 2021; Perez-Barron et al., 2015; García-Alonso et al., 2021).

Preclinical studies have begun to unravel the neuropsychological benefits of HT, particularly in models of stress-induced depression and neurodegeneration. For example, HT delivered by oral gavage to mice subjected to chronic unpredictable mild stress significantly alleviated depressive-like behaviours (Zhao et al., 2021). These effects were accompanied by reduced oxidative stress markers, diminished microglial activation, and enhanced expression of neurotrophic factors in the hippocampus, a key region for synaptic plasticity and emotional regulation. HT can alter the expression of brain receptors and important neuronal plasticity-associated second messenger cascade components, in part, through epigenetic mechanisms including regulation of DNA methylation and downregulation of inhibitory miRNAs (Di Francesco et al., 2015; Zheng et al., 2015a, 2015b). Fan et al. (2023) showed that under conditions of stress-induced blood-brain barrier (BBB) disruption, HT could more easily cross the BBB and preferentially accumulate in the hippocampus, where it modulated lipid metabolism, reduced microglial overactivation, leading to improved behavioural outcomes. Such spatial and mechanistic specificity underscores the potential of HT as a therapeutic for neuropsychiatric conditions.

Further evidence from Li et al. (2024) emphasised the anti-neuroinflammatory effects of HT. Using transcriptomic and metabolomic analyses in rodent models of depression, HT was found to restore mitochondrial function and preserve mitochondrial ultrastructure. HT also reduced inflammation-induced neuronal apoptosis. HT can improve the function of the hypothalamic-pituitary-adrenal (HPA) axis, a central system governing the stress response. Specifically, Arunsundar et al. (ArunSundar et al., 2018) reported that HT restored HPA axis homeostasis and cognitive performance in an Alzheimer's disease (AD) model.

Taken together, these findings suggest that hydroxytyrosol has the potential to address core domains of mental Illness, including mood disturbances, cognitive decline, and social dysfunction. Very little is known about the effect of HT on the latter two behavioural domains, and even less about the molecular and cellular mechanisms by which HT can mediate neuroprotection. There is a pressing need to more fully characterise the transcriptional regulation mediated by HT to better understand the agent's neuroprotective and mental health-promoting properties.

In this study, we investigated the effects of HT on mental resilience in rats exposed to a novel Chronic Unpredictable Restraint Stress (CURS) paradigm. We assessed cognitive, social and affective behaviours using the novel object recognition (NOR), social interaction (SI), sucrose preference test (SPT), open field test (OFT) and elevated plus maze (EPM) tests. Expression levels of key neurotransmitters, dopamine and serotonin, and serum corticosterone were quantified. To examine the molecular basis of the action of HT, we performed single-cell RNA sequencing (scRNA-seq) of the hippocampus.

## 2 Methods

### 2.1 Animals

Postnatal day 54 (P54) female and male Sprague-Dawley rats were obtained from Charles River Laboratories (UK) and housed in the Biomedical Facility, University College Dublin. Animals were acclimatised for one week prior to experimental procedures. Rats were housed in groups of four under controlled temperature (22 °C) and a 12 h light/dark cycle, with *ad libitum* access to standard pellet chow and water. Animal use and experiments were approved by the Animal Research Ethics Committee of UCD (AREC-P-09-23-Murphy) and the Health Products Regulatory Authority (HPRA) (AE18982/P112). All animal work was carried out by individuals who held the appropriate licence issued by the HPRA of Ireland. Animals were given identification numbers and were arbitrarily assigned to the different training/treatment groups.

### 2.2 Hydroxytyrosol preparation and administration

Hydroxytyrosol (HT) was administered orally using a gelatine-based jelly formulation prepared with no-added-sugar cranberry juice. Gelatine was dissolved in cranberry juice under gentle heating, after which HT was added to achieve a daily dose of 50 mg/kg body weight. For example, a 250 g rat received 12.5 mg HT incorporated into 1 mL of jelly. The solution was thoroughly mixed to ensure homogeneity, dispensed into individual moulds, and allowed to set at room temperature. Solidified jellies were stored at 4 °C until use.

HT content in representative jellies was confirmed by HPLC as described by Davis et al. (2018). Individual jellies were melted at 50 °C, diluted in deionised water, filtered (Whatman Mini-UniPrep™, 0.45 μm), and analysed using a 20 μL injection volume.

HT was administered once daily at 08:00 h (lights on at 07:00 h). All animals routinely consumed the full jelly within 5 min. Control animals received identical jelly formulations without HT. The HT source was 1-HT® hydroxytyrosol (>98 % purity; Nova Mentis Ltd., Dublin, Ireland). Explicitly, the four treatment groups were non-stressed control diet (NS-CD), non-stressed hydroxytyrosol (NS-HT), restraint stress control diet (RS-CD) and restraint stress hydroxytyrosol (RS-HT).

### 2.3 Chronic unpredictable restraint stress (CURS)

Restraint stress was applied using custom acrylic cylinders (6.5 cm inner diameter, 20 cm length) with nasal ventilation ports. Animals were placed gently into the cylinders, allowing restricted movement without compression. Stress exposure occurred daily between P61 and P74, commencing at 09:00 h. Stress duration was predetermined and varied pseudo-randomly between 0 and 6 h per day in a non-repeating pattern to reduce predictability and habituation (Table 1). Non-stressed control animals were handled daily for 5 min between 09:00 and 10:00 h.

**Table 1:** Duration of the restraint on each day of the CURS paradigm.

Day	1	2	3	4	5	6	7	8	9	10	11	12	13	14
Stress Duration (hrs)	6	6	0	6	2	6	4	6	2	6	4	6	4	6

### 2.4 Behavioural testing

Behavioural assessments were conducted from P75 to P78 following completion of CURS. HT treatment continued daily. Tests were selected to assess anhedonia, anxiety-like behaviour, recognition memory, and sociability. All behavioural testing was performed in a temperature- and light-controlled behavioural suite during the light phase (09:00–16:00 h). Testing was conducted by the same experimenter under identical conditions. Unless otherwise stated, behaviour was recorded and analysed using an overhead video camera linked to EthoVision XT (v15.0; Noldus, UK) tracking software. Data were exported to GraphPad Prism 10 for statistical analysis.

#### 2.4.1 Sucrose preference test

Anhedonia was assessed in home cages using the sucrose preference test as described by Willner et al. (1987) with minor modifications. On P56, animals were habituated to two drinking bottles containing water for 24 h. On each of P57-59, one of the water bottles was replaced with a 1% sucrose solution for 1 h. Following overnight food and water deprivation, on P60 and P75, animals were given simultaneous access to water and 1% sucrose for 2 h, with bottle positions switched after 1 h. Intake was determined gravimetrically. Sucrose preference was calculated as a percentage:

Sucrose solution intake (mL)/Total fluid intake (mL) x 100

#### 2.4.2 Open field test

Locomotor activity was assessed in an open field arena (65 × 65 × 40 cm, black acrylic). Animals were placed in the centre of the arena and allowed to explore freely for 5 min. EthoVision XT quantified total distance travelled, mean velocity, and time spent in the centre zone (25 × 25 cm) relative to the periphery.

#### 2.4.3 Elevated plus maze

Anxiety-like behaviour was assessed using the elevated plus maze as described by Handley and Mithani (1984) with minor modifications. The maze comprised two open arms (50 × 10 cm) and two closed arms (50 × 10 cm, 40 cm walls) elevated 100 cm above the floor. Animals were placed at the central junction facing an open arm and allowed to explore for 5 min under dim lighting. EthoVision XT quantified time spent and entries into open and closed arms. The percentage of time spent on and entries into open arms were used as primary indices of anxiety.

#### 2.4.4 Novel object recognition

Recognition memory was assessed using the novel object recognition task. Animals were habituated to the empty arena (65 × 65 × 40 cm, black acrylic) for 5 min. Twenty-four hours later, two identical objects were presented for 5 min (training session). After 1 h back in their home cage, animals were returned to the arena where one object was replaced with a novel object and exploration was recorded for 5 min. Object identity and position were counterbalanced across animals of a particular group. Object exploration was scored by an observer and defined as nose-oriented investigation within 2 cm of the object. The novel object discrimination index was calculated as a percentage:

Time exploring novel object/Total time exploring either object x 100

#### 2.4.5 Social interaction

Social behaviour was assessed using a three-chamber social interaction paradigm adapted from File and Hyde (1978). Each side chamber was divided into two sub-chambers by a clear Perspex wall with air holes to allow free passage of odours. The test animal could explore the middle chamber and the two side sub-chambers, but the clear Perspex prevented access to the two side sub-chambers at the extreme end of the apparatus. Following a 5 min habituation period and a 5 min timeout, test animals were placed back into the middle chamber of the apparatus, where a novel conspecific was placed in one of the extreme side sub-chambers while the opposite extreme sub-chamber remained empty. The test animal was allowed to explore for 5 min. EthoVision XT quantified time spent in each chamber, while time engaged in social interaction with the conspecific animal was scored by an observer blind to the treatment groups. Sub-chamber positioning of the conspecific animal was counterbalanced, and the apparatus was cleaned between trials.

### 2.5 Sample collection

Following euthanasia, whole blood was collected into 20 mL tubes, allowed to clot for 30 min on ice, and centrifuged at 2000 × g for 10 min at 4 °C. Serum was aliquoted and snap-frozen in liquid nitrogen before storage at −80 °C. Prefrontal cortex and hippocampal tissues were rapidly dissected and snap-frozen. Procedures were identical to those described in Abdulmalek et al. (2024).

### 2.6 Serum corticosterone

Serum corticosterone concentrations were measured using a commercial ELISA kit (Cat. No. ER0859; Wuhan Fine Biological Technology). Samples and standards were assayed in duplicate according to the manufacturer's instructions. Absorbance was measured at 450 nm and concentrations calculated from the standard curve. Results are expressed as pg/mL.

### 2.7 Brain monoamine quantification

Hippocampal and prefrontal cortex tissues were homogenised in RIPA buffer containing protease and phosphatase inhibitors. Dopamine and serotonin levels were quantified using commercial ELISA kits (Elabscience Biotechnology) according to the manufacturer's protocols. Absorbance was measured at 450 nm and concentrations expressed as ng/mL.

### 2.8 Single-cell RNA sequencing

Single-cell RNA sequencing was performed on hippocampal tissue using the 10x Genomics Chromium Nuclei Isolation Kit with RNase inhibitor. We selected the hippocampus for transcriptomic profiling because it is strongly implicated in stress hormone sensitivity, cognitive impairment, mood regulation, and neuroplasticity. Additional dissected tissues, including nucleus accumbens, amygdala, hypothalamus, and midbrain regions, were retained for future region-specific analyses. For each treatment group, the middle third of the left hippocampus of each of 3 animals was pooled into a single sample and processed to obtain a single cell suspension. This was done separately for males and females in each treatment group yielding 6 samples. Isolated cells were encapsulated into gel bead-in-emulsions using the Chromium Controller and processed using the Chromium Next GEM Single Cell 3’ Kit v3.1. Libraries were quality-controlled and sequenced on an Illumina platform.

Reads were aligned to the rat genome (Rnor_6.0) using CellRanger v7.2.0 (10x Genomics). Downstream analysis was performed in Seurat v5.0 (Satija et al., 2015). Cells with less than 200 detected genes, greater than 2500 genes (to remove doublets) or greater than 10 % mitochondrial transcripts were excluded. Following this quality filter, a total of 148,369 cells were retained across all samples (see Fig. S6 for numbers of cells per group). Data were normalised and integrated using the SCTransform function in Seurat. Clustering was performed using PCA and UMAP. Cell types were assigned using canonical markers. Differential expression analysis was performed using Seurat FindMarkers with a Wilcoxon rank-sum test. Raw p-values were adjusted for multiple comparisons using the Benjamini–Hochberg false discovery rate procedure within each comparison. Genes with adjusted P < 0.05 and |log_2_ FC| ≥ 0.3 were considered significantly differentially expressed. Gene Ontology (GO) and Kyoto Encyclopedia of Genes and Genomes (KEGG) enrichment analyses were conducted using clusterProfiler v4.10 (Wu et al., 2021).

### 2.9 Statistical analysis

Other than the single cell analyses described above, statistical analyses were performed using GraphPad Prism. Behavioural and biochemical data were analysed using two-way ANOVA followed by Tukey's post-hoc test where appropriate. Statistical significance was set at P < 0.05.

## 3 Results

Chronic unpredictable restraint stress (CURS) slightly blunted the normal weight gain observed in female and male rats over the two-week restraint period (Fig. S1). The hydroxytyrosol (HT) diet did not alter this stress effect in female rats but partially alleviated it in male animals.

### 3.1 HT prevents CURS-induced anxiety and depressive-like behaviours

**Fig. 1:**
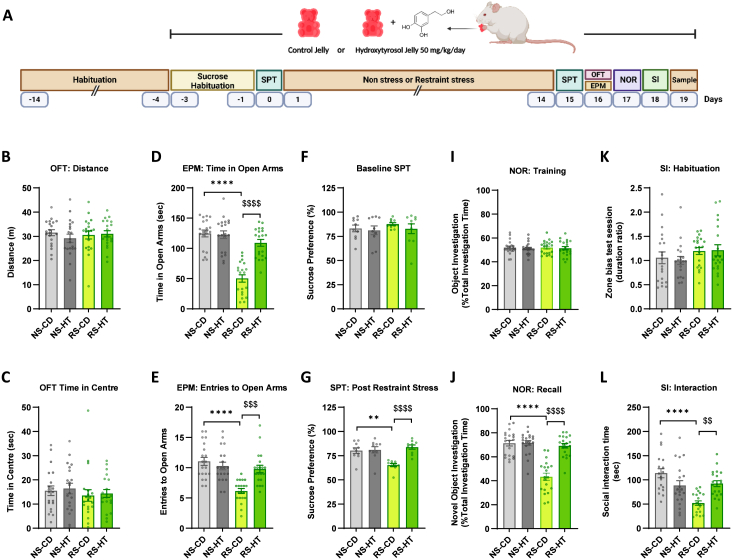
**Effects of restraint stress and HT on behaviours relevant to anxiety, depression and cognitive function. A:** Experimental design for the behavioural animal cohort. **B, C:** Open-field test (OFT) showing total distance travelled and time spent in the centre, respectively, during a 5-min session. **D, E:** Elevated plus maze (EPM) performance, represented by time spent in (**D**) and entries into (**E**) open arms. **F, G:** Sucrose preference test (SPT) assessing anhedonia before and after restraint stress, respectively. **I, J:** Novel Object Recognition (NOR) test assessing working memory. For the recall test, this data is presented as the percentage of total investigation time spent on the novel object during the test session. In the training session, both objects are the same. **K, L:** Social Interaction (SI) paradigm assessing social behaviour. **K:** Habituation phase; **L:** total social interaction time with novel stimulus animal. Data are mean ± SEM (n = 20, 10 male and 10 female animals per group). Individual animals are represented by small circles. ∗∗P < 0.01, ∗∗∗∗P < 0.0001 vs. control (NS-CD); ^$$^ P < 0.01, ^$$$^ P < 0.001, ^$$$$^ P < 0.0001 vs. CURS animals (RS-CD) (two-way ANOVA with Tukey's post hoc test). NS-CD: non-stressed control diet; NS-HT: non-stressed hydroxytyrosol; RS-CD: restraint stress control diet; RS-HT: restraint stress hydroxytyrosol.

The experimental design for the behavioural animal cohort is shown in Fig. 1A. Animals were placed on either the control or HT diet prior to exposure to the chronic unpredictable restraint stress (CURS) procedure. Non-stressed animals were handled for 5 min and then remained in their home cage as controls for the stress intervention. In the open field test, no significant differences were observed in total distance travelled or time spent in the centre zone between control (NS-CD), CURS (RS-CD), and HT-treated groups (NS-HT & RS-HT; Fig. 1B and C). These results indicate that neither restraint stress nor HT influenced general locomotor activity.

Anxiety-like behaviour was evaluated using the elevated plus maze (Fig. 1D and E). Consistent with the induction of an anxiety phenotype, CURS animals on a control diet (RS-CD) spent significantly less time in the open arms compared to non-stressed controls (NS-CD; ∗∗∗∗P < 0.0001), and made fewer open-arm entries. HT treatment normalised both the time spent in open arms and the open-arm entries of CURS-treated animals. For this and subsequent behavioural datasets, analysis with sex included as a factor revealed no significant sex × treatment interactions for the behavioural outcomes. For that reason, data combining both sexes are presented in the main figures, with sex-disaggregated data provided in Supplementary Fig. S2–S5.

To assess depressive-like behaviours, we used the sucrose preference test (SPT), a test of anhedonia. At baseline, tested prior to the commencement of the restraint stress paradigm, there were no significant differences between groups, with all groups showing a strong preference for sucrose over water (Fig. 1F). The CURS exposure group (RS-CD) showed a significant reduction in sucrose preference compared to controls (NS-CD; Fig. 1G; ∗∗P < 0.01). HT treatment effectively reversed this effect, with CURS + HT animals (RS-HT) demonstrating significantly higher sucrose preference (^$$$$^ P < 0.0001) than their CURS counterparts. Again, these effects of stress and HT were observed in both female and male animals (Fig. S2).

### 3.2 HT rescues stress-impaired recognition memory

In the novel object recognition test of working memory cognitive function, all animals showed no object location bias during the training session with two identical objects (Fig. 1I). During the memory test, the control animals exhibited robust recognition of the novel object as revealed by the bias to that object during object exploration (Fig. 1J). CURS-treated rats displayed deficits with a significantly reduced percentage of time spent exploring the novel object (∗∗∗∗P < 0.0001). In fact, CURS animals showed no exploration bias at all, suggesting that they had no recall of the familiar object or recognition of the novel object. HT treatment significantly improved overall performance (^$$$$^ P < 0.0001) and in female (^$$$$^ P < 0.0001) and male (^$$^ P < 0.01) animals, specifically (Fig. 1J; Fig. S3).

### 3.3 HT protects against stress-induced deficits in social interaction

In the acclimatisation session, in the absence of a stimulus animal, all animal groups spent equal time in the interaction and avoidance zones of the apparatus, indicating no zonal bias that could confound interpretation of the social interaction test (Fig. 1K). In the social interaction (SI) session, CURS animals spent significantly less time engaging with a novel conspecific than their non-stressed control counterparts, indicative of a social withdrawal phenotype (Fig. 1L; ∗∗∗∗P < 0.01). HT treatment significantly increased interaction time (^$$^ P < 0.01), suggesting a protective role in maintaining social behaviour (Fig. 1L). Again, these effects were seen in both male and female animals (Fig. S3).

### 3.4 HT mitigates stress-induced hypercorticosteronaemia

**Fig. 2:**
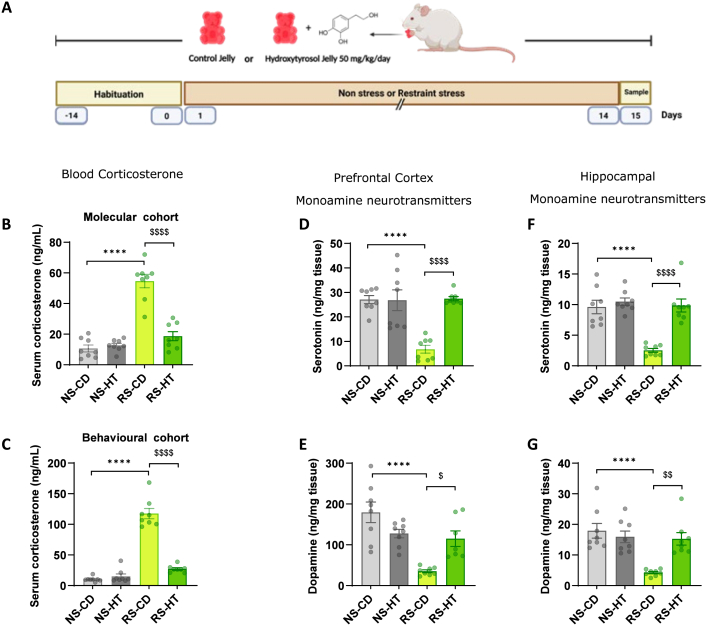
**HT protects against stress-induced alterations in stress responsivity, monoamine neurotransmitter levels and neuroplastic protein levels. A:** Experimental design for the molecular cohort. **B, C:** Serum corticosterone levels in the molecular and behavioural cohorts, respectively. **D, E:** Effect of CURS and HT on prefrontal serotonin and dopamine levels, respectively. **F, G:** Effect of CURS and HT on hippocampal serotonin and dopamine levels, respectively. Data are presented as mean ± SEM (n = 8 (4 females, 4 males) per group; circles represent individual animals). Statistical comparisons were performed using two-way ANOVA with Tukey's post hoc test. NS-CD vs RS-CD: ∗∗∗∗P < 0.0001; RS-CD vs RS-HT: ^$^ P < 0.05, ^$$^ P < 0.01, ^$$$$^ P < 0.0001. NS-CD: non-stressed control diet; NS-HT: non-stressed hydroxytyrosol; RS-CD: restraint stress control diet; RS-HT: restraint stress hydroxytyrosol.

We included a parallel animal cohort (Fig. 2A), which did not undergo behavioural testing, in order to harvest brain tissue and blood serum for biochemical and neurochemical analyses that would not be confounded by the conduct of the behavioural battery.

The level of corticosterone in the blood is a key indicator of stress. In the serum samples collected, unsurprisingly, CURS significantly elevated serum corticosterone (Fig. 2B; ∗∗∗∗P < 0.0001). HT treatment robustly protected against this effect, supporting its role in HPA axis normalisation. Interestingly, the hypercorticosteronaemia was even greater when measured in the behavioural cohort, in serum samples gathered 24h after the final behavioural test (Fig. 2C). This would suggest that the CURS group show an exaggerated stress response to the experience of the novel behavioural testing environments. Significantly, therefore, HT is protective even against this hypersensitive stress response (Fig. 2C). These effects were evident in both sexes (Fig. S4).

### 3.5 HT preserves serotonin and dopamine levels in the brain

Serotonin and dopamine are two neurotransmitters that are central to disorders of mental health, such as anxiety and depression (Krishnan and Nestler, 2008; Jiang et al., 2022). Neurochemical analyses revealed that CURS significantly decreased serotonin and dopamine levels in both the prefrontal cortex and hippocampus (Fig. 2D–G; Fig. S5). HT treatment protected serotonin and dopamine levels from this stress-induced decline across both brain structures and sexes.

### 3.6 HT effects on CURS-induced transcriptional dysregulation in the hippocampus

**Fig. 3:**
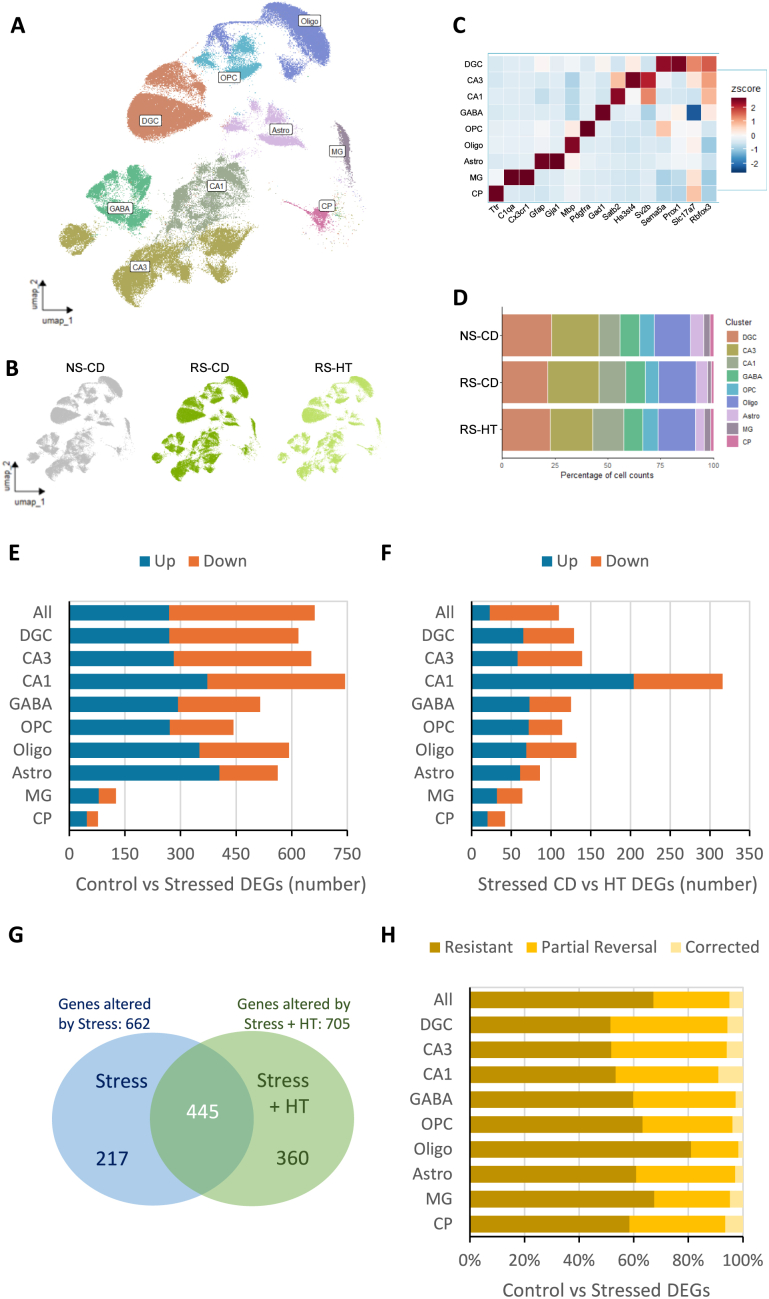
**Single cell RNAseq transcriptional analysis of the effect of stress and HT. A:** UMAP showing cell type clusters. **B:** umap split by the control, stressed and stressed & HT samples. **C:** Cell type marker genes heatmap. **D:** Cell type proportions per treatment group. **E:** Number of genes dysregulated by chronic unpredictable restraint stress overall (All) and in each cell type. **F:** Number of genes regulated by HT in chronic unpredictable restraint stress animals overall (All) and in each cell type. **G:** Overlap between stress-associated DEGs (NS-CD (control) vs RS-CD) and DEGS when comparing NS-CD vs RS-HT). **H:** Proportion of Stress-associated transcriptional dyregulation resistant to, partially reversed by (no longer different between NS-CD and RS-HT), or corrected by HT (meet criterion for partial reversal and are significantly different between NS-CD and RS-CD). DEG: adj_p_value < 0.05 and |log2FC| ≥ 0.3. NS-CD: non-stressed control diet; RS-CD: restraint stress control diet; RS-HT: restraint stress hydroxytyrosol; DGC: dentate granule cells; CA3: CA3 pyramidal neurons; CA1: CA3 pyramidal neurons; GABA: GABAergic interneurons; OPC: Oligodendrocyte precursor cells; Oligo: Oligodendrocytes; Astro: astrocytes; MG: microglia; CP: choroid plexus epithelial cells.

Following quality control filtering, single-cell RNA sequencing yielded transcriptional profiles from 148,369 hippocampal cells (Fig. 3A and B; Fig. S6). Based on the expression of well-established cell-type marker genes (Fig. 3C), all the major neural and glial populations in this brain structure were present, including dentate granule cells, CA1 and CA3 pyramidal neurons, GABAergic interneurons, astrocytes, oligodendrocytes, oligodendrocyte precursor cells, microglia, and choroid plexus epithelial cells (Fig. 3A–D).

### 3.7 Transcriptional fingerprint of the chronic unpredictable restraint stress (CURS) model

Using pseudobulk analysis, exposure to CURS induced extensive transcriptional dysregulation, with 662 genes significantly altered in the hippocampus compared with controls (adjusted P < 0.05, |log_2_ FC| ≥ 0.3; Fig. 3E). The greatest magnitude of stress-induced transcriptional change occurred within CA1 pyramidal neurons, but substantial dysregulation was evident in most cell types present, including neuronal and non-neuronal cell types (Fig. 3E). In contrast, in the context of the CURS model, HT treatment altered the expression of 110 genes overall (Fig. 3F). CA1 pyramidal neurons appear particularly sensitive to HT treatment, exhibiting a far great magnitude of gene expression response compared with all other cell types (316 genes; Fig. 3F). Approximately 30% of the stress-responsive genes (NS-CD vs RS-CD) were not differentially expressed in stressed animals receiving hydroxytyrosol (NS-CD vs RS-HT; Fig. 3G and H). On average, neuronal cell types (DGC, CA3, CA1, GABA) exhibited a better HT-mediated correction (40% stress-associated gene changes no longer different) compared with non-neuronal cell types at 30% (OPC, Oligo, Astro, MG; Fig. 3H).

**Fig. 4:**
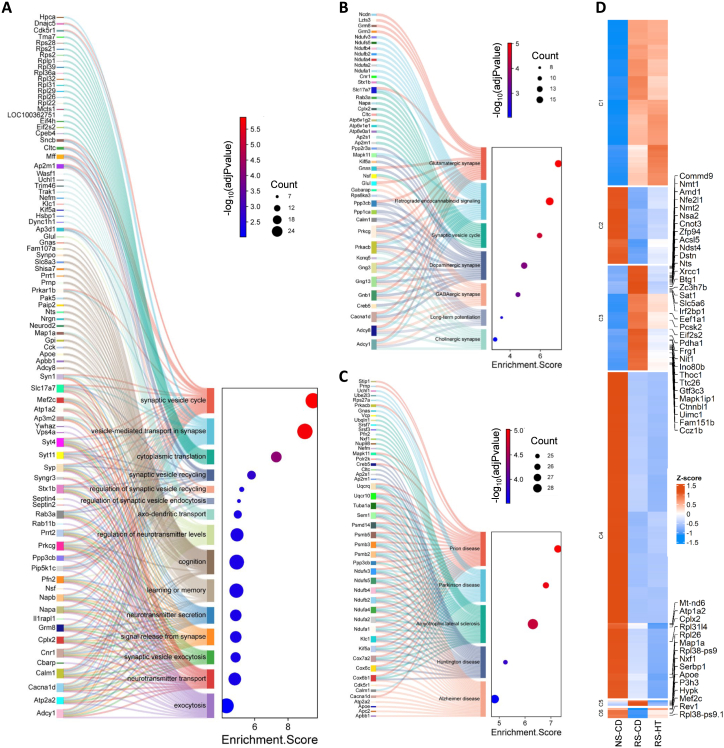
**Single cell RNAseq transcriptional analysis of the effect of stress. A:** Sankey & dot plots showing DEGs among the top 30 most significant stress-associated GO: Biological Functions related to higher cognition and pre-synaptic vesicle function dysregulated by CURS. **B:** Stress-dysregulated KEGG Pathway analysis related to synaptic transmission across the major neurotransmitters. **C:** Stress-dysregulated KEGG Pathway analysis clearly implicates neurodegenerative processes. DEG: adj_p_value < 0.05 and |log2FC| ≥ 0.3. **D:** Hierarchical clustering of stress-regulated DEGs identifies different patterns of dysregulation. Genes corrected by HT are indicated by name. NS-CD: non-stressed control diet; RS-CD: restraint stress control diet; RS-HT: restraint stress hydroxytyrosol.

Functional enrichment analyses of the stress-regulated gene set revealed that stress predominantly disrupted pathways linked to synaptic transmission, vesicle trafficking, calcium-dependent exocytosis, mitochondrial oxidative phosphorylation, and ribosomal translation (Fig. 4A; Fig. S7). KEGG pathway mapping further implicated dysregulation across glutamatergic, GABAergic, dopaminergic, and cholinergic synapses, with significant enrichment of neurodegenerative-disease modules including Alzheimer's, Parkinson's, and prion disease pathways (Fig. 4B and C). Hierarchical clustering of stress-associated DEGs clearly identified HT-resistant stress responses (clusters 1, 2, 4) and HT-responsive stress-regulated genes (clusters 3, 5 and 6; Fig. 4D).

#### 3.7.1 HT reversal of CURS-induced hippocampal gene expression change

**Fig. 5:**
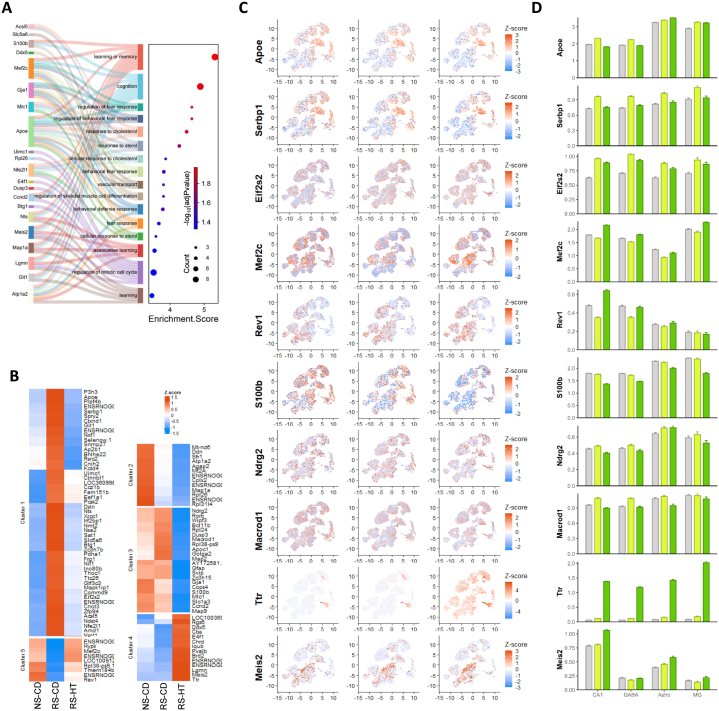
**Single cell RNAseq transcriptional analysis of effect of HT on stress-associated dysregulation. A:** Sankey & dot plot showing DEGs among the top 20 most significant GO: Biological Functions regulated by HT in CURS animals. **B:** Hierarchical clustering of HT-regulated DEGs (RS-CD vs RS-HT) identifies different patterns of dysregulation. **C:** Exemplar gene expression UMAPs for genes upregulated by stress and corrected by HT (*Apoe, Serbp1, Eif2s2*), genes downregulated by stress and corrected by HT (*Mef2c, Rev1*), genes downregulated by HT independent of stress (*S100b, Ndrg2, Macrod1*), and genes upregulated by HT independent of stress (*Ttr, Meis2*). **D:** Violin plots showing expression of the same exemplar HT-regulated DEGs across CA1 pyramidal excitatory neurons (CA1), GABAergic interneurons (GABA), astrocytes (Astro) and microglia (MG). NS-CD: non-stressed control diet; RS-CD: restraint stress control diet; RS-HT: restraint stress hydroxytyrosol.

To better understand the protective action of hydroxytyrosol against stress-induced anxiety, depression and cognitive dysfunction, we next analysed the genes significantly regulated by HT in the CURS model (Fig. 5). GO pathway analysis demonstrated significant enrichment for genes involved in higher cognitive functions like learning and memory and stress/fear responsivity (Fig. 5A), suggesting HT supports these functions preserving them against stress-induced dysregulation. Moreover, KEGG pathways enriched by the HT-regulated genes implicate modulation of nucleocytoplasmic transport, synaptic vesicle cycling, ferroptosis and cholesterol metabolism, in particular, in the protective action of HT (Fig. S8). Hierarchical clustering revealed gene groups where HT is reversing the effects of stress (clusters 1 and 5; Fig. 5B) and clusters where HT is regulating gene expression independently of stress (clusters 3 and 4). Among the stress-associated up-regulated genes normalised by HT (cluster 1), there was a coordinated regulation of genes associated with glial reactivity, cellular stress responses, and transcriptional and translational regulation. For example, expression of *Apoe*, a marker of stress- and injury-associated astrocytic and microglial states (Zhang et al., 2016; Krasemann et al., 2017), was significantly increased by stress. Similarly, chronic stress elevated hippocampal levels of genes such as Serbp1, an RNA-binding protein involved in the regulation of mRNA metabolism (Ji and Ablimit, 2025; Lee et al., 2014), and Eif2s2, a core component of the eIF2 translation initiation complex. Administration of HT normalised the expression of *Apoe, Serbp1* and *Eif2s2* toward control levels (Fig. 5C and D). Conversely, in cluster 5, *Mef2c,* a key activity-regulated transcription factor important for synapse maturation and stability in the hippocampus, is downregulated in the stressed animals but then corrected by HT (Fig. 5C and D).

#### 3.7.2 Stress-independent HT enhancement of hippocampal neuroplasticity and resilience

Within the non-stress regulated genes modulated by HT (clusters 3 and 4), a triad of genes quintessentially associated with neuroinflammation, *Gfap, Gja1* and *S100b*, were all down-regulated by HT (Fig. 5C and D). Another gene sharing this pattern of regulation was *Ndrg2*, an astrocyte-enriched gene implicated in stress-induced astrocytic remodelling and impaired neuronal support (Wang et al., 2025). Interestingly, there is a concordant HT-mediated upregulation of *Meis2* (Fig. 5C–D), a developmental transcription factor that promotes neuronal fate and can suppress glial programs, that would include genes such as *Gfap, Gja1* and *S100b* (Agoston et al., 2014; Schulte and Geerts, 2019). *Meis2* is linked to *Ttr*, another neuroplasticity-associated gene (Monopoli et al., 2011) upregulated by HT (Fig. 5C and D), through retinoic acid biology, as *Ttr* transports retinol, key to the synthesis of retinoic acid, a strong activator of *Meis2* (Blaner, 2019b; Vitobello et al., 2011). HT also upregulated the BMP inhibitor chordin (*Chrd*), which promotes hippocampal neurogenesis and neuroplasticity essential for normal cognitive functions such as mood regulation, learning and memory (Bond et al., 2012; Gobeske et al., 2009).

## 4 Discussion

The health benefits of the Mediterranean diet have long been appreciated, particularly for its protective effects on brain health and its potential to reduce the risk of neurodegenerative and mental health conditions (Kiani et al., 2020). The positive influence of olive oil-derived polyphenols, and hydroxytyrosol (HT) in particular, on brain function has been well established, including enhancements in neurogenesis, cognitive abilities such as memory and learning, and overall neural protection (Leri et al., 2020; Angeloni et al., 2017). In the present study, we demonstrate that biotechnologically produced HT confers robust protection against the behavioural and molecular effects of chronic unpredictable restraint stress (CURS) in rats.

Our novel version of CURS induced behavioural phenotypes that mimic core features of mood disorders, including anhedonia, anxiety, cognitive impairment, and social withdrawal. The novel use of an abbreviated two-week variable-duration restraint paradigm produces robust behavioural and endocrine stress phenotypes within a shorter timeframe than many conventional chronic stress protocols. This has important implications for the field as it allows a much shorter duration of stress on the animals and represents an important refinement from an animal welfare perspective. Moreover, compared with acute assays such as the forced swim test, the CURS paradigm offers stronger construct validity because it models prolonged and unpredictable stress exposure (Markov and Novosadova, 2022). It also captures a wider behavioural phenotype, including anhedonia, anxiety, cognition and social withdrawal, and is more suitable for evaluating preventative or chronic treatment strategies (Commons et al., 2017; Yankelevitch-Yahav et al., 2015).

Treatment with HT protected against the effects of CURS across all behavioural domains measured, suggesting its capacity as a broad-spectrum mental resilience agent (Willner, 2005; Cicerale et al., 2010, 2012; Zaletel et al., 2017). Importantly, this was achieved through the novel use of jelly-based precise oral administration, permitting individual dosing without the repeated handling and restraint associated with gavage or the inevitable inaccuracy of dosing via *ad libitum* chow or water. HT-treated CURS rats maintained normal sucrose preference, spent the usual amount of time in the open arms of the elevated plus maze, and exhibited normal social interaction, all behaviours disrupted by CURS in untreated chronically-stressed animals. Moreover, HT also protected against CURS impairment of recognition memory. The observed anxiolytic and antidepressant effects of HT are consistent with previous reports highlighting the neuroprotective benefits of olive oil-derived polyphenols (Kiani et al., 2020; Leri et al., 2020; Zhao et al., 2021; Valls-Pedret et al., 2015; Yoon et al., 2023; Angeloni et al., 2017) while the positive effects on working memory and social interaction, additionally suggest robust support of cognitive function and social connectedness, respectively.

The lack of effect of HT on OFT locomotion indicates that HT did not modify general motor activity under the present experimental conditions. Unchanged OFT performance supports the view that the antidepressant, anxiolytic and neuroprotective effects of HT are not attributable to nonspecific psychostimulant or sedative actions.

CURS exposure led to substantial reductions in hippocampal and cortical serotonin and dopamine levels, neurotransmitters critically implicated in affective regulation and reward processing (Krishnan and Nestler, 2008; Jiang et al., 2022). HT administration effectively prevented these reductions, indicating protection of monoaminergic circuitry. The preservation of dopamine and serotonin observed here may reflect multiple convergent mechanisms. In addition to limiting oxidative and inflammatory stress, hydroxytyrosol has been reported to reduce monoamine breakdown through inhibition of the monoamine degradation enzymes MAO and COMT (Gallardo et al., 2015; Perez-Barron et al., 2021b), which may contribute to maintenance of neurotransmitter availability under chronic stress conditions.

HT prevented stress-induced rises in serum corticosterone levels, indicating maintenance of a normal HPA axis homeostasis. Chronic hypercorticosteronaemia is well-established to disrupt hippocampal function and contributes to depressive phenotypes (Sapolsky et al., 1986), and so the stabilisation observed in HT-treated CURS animals further underscores a role for HT in stress resilience. Interestingly, serum corticosterone levels were even higher in stressed animals subjected to behavioural testing, suggesting sensitisation to environmental stressors. The capacity of HT to blunt even this exaggerated stress response further highlights its potential as a beneficial modulator of stress reactivity. Marked hypercorticosteronaemia together with convergent behavioural impairments support the face validity of the current CURS procedure. Moreover, the present study extends previous HT literature by combining multilevel behavioural phenotyping with endocrine, neurochemical and single-cell transcriptomic analyses in both female and male rats, thereby providing a more comprehensive mechanistic framework for HT-mediated stress resilience.

### 4.1 Novel chronic unpredictable restraint stress (CURS) paradigm disrupts synaptic transmission, mitochondrial function, and protein translation

Consistent with previous large-scale transcriptomic studies of stress and glucocorticoid exposure (Chen et al., 2022; Szyf, 2021), CURS induced robust dysregulation of genes critical for synaptic transmission (e.g. *Nsf, Syn1, Syt4*), mitochondrial oxidative phosphorylation (e.g. *Ndufb2, Cox6c*), and ribosomal biogenesis (multiple *Rps* and *Rpl* genes). These pathways are essential for sustaining neuronal energy metabolism and synaptic efficacy, processes that are particularly vulnerable to prolonged stress-associated glucocorticoid signalling. Single-cell resolution revealed that transcriptional perturbations were most pronounced in excitatory glutamatergic neurons of the hippocampus, including CA1 and CA3 pyramidal neurons and dentate granule cells, cell types known to undergo dendritic atrophy and spine loss following chronic stress (Zaletel et al., 2017; Duman and Aghajanian, 2012). Significant alterations were also evident in GABAergic interneurons and across glial populations, revealing a system-wide impact of chronic stress on hippocampal circuitry.

CURS was associated with dysregulation of genes with established roles in learning, memory, and higher cognitive function, as well as with altered signalling for most of the major neurotransmitter systems. This broad disruption likely contributes to excitation:inhibition imbalance and aberrant neuromodulation by acetylcholine and dopamine, which might, in turn, underpin deficits in memory encoding and cognitive flexibility (Tsetsenis et al., 2022; Pérez-González et al., 2024; Chen et al., 2022). In parallel, pathway-level analyses revealed enrichment for gene signatures associated with multiple neurodegenerative disorders, including prion disease, Parkinson's disease, Alzheimer's disease, Huntington's disease, and amyotrophic lateral sclerosis. These findings strongly implicate mitochondrial stress, impaired protein synthesis, and proteasomal dysfunction as core mechanisms through which chronic stress compromises the hippocampal circuits governing cognition, mood regulation, and stress responsivity. Notably, the overlap between stress-induced and ageing-related transcriptomic changes reported here aligns with prior observations in both animal and human studies (Wang et al., 2023; Szyf, 2021).

### 4.2 HT prevents stress-induced molecular and cellular dysregulation in the hippocampus

HT prevented dysregulation of a large subset of CURS-associated transcriptional changes, preserving expression of genes involved in presynaptic vesicle cycling, mitochondrial respiration, oxidative stress defence, and protein homeostasis. These effects suggest that HT buffers hippocampal cells against chronic stress by maintaining energetic capacity and limiting oxidative and metabolic strain. Consistent with this interpretation, similar protective effects on mitochondrial structure and oxidative phosphorylation have been reported in rodent models treated with HT (Li et al., 2024; Zheng et al., 2015a, 2015b). Our data demonstrate, for the first time, that chronic unpredictable restraint stress (CURS) elicits a cell-type-specific cascade of molecular dysfunction and that HT broadly mitigates these deficits across neuronal and non-neuronal populations.

HT normalised expression of *Mef2c*. In the hippocampus, *Mef2c* functions as an activity-dependent transcription factor, translating neuronal activity into long-term gene expression programs to shape circuit stability and plasticity (Flavell et al., 2006, 2008). For example, loss of *Mef2c* impairs hippocampal-dependent contextual fear memory and spatial learning (Barbosa et al., 2008), while in humans, *Mef2c* haploinsufficiency causes severe intellectual disability (Le Meur et al., 2010; Paciorkowski et al., 2013). Therefore, the preservation of *Mef2c* expression may represent a key mechanism by which HT supports synaptic homeostasis and hippocampal-dependent cognition. HT also prevented decreased expression of *Rev1*, a specialised DNA polymerase important for DNA repair (Sale et al., 2012; Madabhushi et al., 2014). Together, these findings suggest that the neuroprotective effects of HT extend beyond antioxidant activity to encompass preservation of transcriptional and translational programs essential for synaptic maintenance and cognitive function.

At the molecular level, CURS drove coordinated upregulation of genes such as *Apoe, Serbp1,* and *Eif2s2*, collectively indicative of glial activation and altered translation. Elevated *Apoe* is a recognised marker of stress- and injury-associated glial states linked to synaptic destabilisation (Zhang et al., 2016; Krasemann et al., 2017). *Serbp1* encodes an RNA-binding protein whose roles in stress granule dynamics and RNA regulation suggest it could modulate neuronal and glial translational responses under prolonged stress (Ji and Ablimit, 2025; Lee et al., 2014). Upregulation of *Eif2s2* points to engagement of the integrated stress response (Costa-Mattioli et al., 2005; Stewart, 2010; Wickramasinghe and Laskey, 2015). Normalisation of these genes by HT further reveals preservation of transcription and translation and protection against neuroinflammation and chronic cellular stress-induced deficits in synaptic function.

### 4.3 HT enhances neuroplasticity and dampens neuroinflammation

Independent of stress-associated dysregulations, HT actively promoted transcriptional programs associated with resilience and plasticity. For example, HT increased the expression of *Brd2*, a BET-family chromatin reader linking histone acetylation to activity-dependent transcription (Denis et al., 2006; Korb et al., 2015). HT also raised *Pvalb* expression, a key marker of fast-spiking interneurons essential for network synchrony and cognitive function (Hu et al., 2014). Chronic stress–related reductions in *Pvalb* have been linked to impaired inhibitory control and disrupted excitation:inhibition balance (Czéh et al., 2015; Page et al., 2019), suggesting that HT promotes network-level stability through increased *Pvalb* expression.

Within stress-independent HT-regulated gene clusters, HT reduced the expression of *Gfap, Gja1, Ndrg2* and *S100b*, genes indicative of neuroinflammation. For example, *Ndrg2* is an astrocyte-enriched gene that is consistently upregulated in the hippocampus following chronic stress, where it has been linked to astrocyte dysfunction and depressive-like phenotypes (Araya-Callís et al., 2012; Wang et al., 2025).

Conversely, HT upregulated *Meis2, Ttr* and *Chrd*. *Meis2* promotes neuronal differentiation, suppresses glial programs (Agoston et al., 2014; Schulte and Geerts, 2019) and is activated by *Ttr*-transported retinoic acid (Blaner, 2019a). *Ttr* (transthyretin), in turn, is substantially produced in the choroid plexus and cooperates with *Chrd* to support hippocampal neurogenesis and plasticity (Bond et al., 2012; Gobeske et al., 2009; Monopoli et al., 2011). These coordinated changes suggest that HT not only counteracts stress-induced degenerative neuroinflammation but also fosters a transcriptional environment that supports cognition-associated neuroplasticity, embedding resilience against the detrimental effects of chronic stress.

In summary, our multiscale analyses demonstrate that chronic stress induces cell-type-specific transcriptional vulnerabilities across the hippocampus. These vulnerabilities include compromised mitochondrial metabolism, oxidative stress defence, protein synthesis, and proteasomal function, with downstream consequences for synaptic plasticity and higher cognitive processes. Hydroxytyrosol confers robust resilience by preserving these core cellular systems, restoring coordinated transcriptional and translational control, and stabilising synaptic network function. Moreover, HT creates a pro-neuroplastic and anti-neuroinflammatory environment that would clearly underpin long-term protection of neural function. Together, these findings provide a molecular framework for dietary HT modulation of stress-related neurobiology. Given the overlap between chronic stress biology and neurodegenerative processes, HT may have translational relevance in disorders such as Parkinson's disease and Alzheimer's disease, where affective symptoms and cognitive decline frequently coexist. The current study positions HT as a promising, biotechnologically producible intervention for enhancing cognitive resilience and preserving mental health under chronic stress.

## Ethical approval statement

All animal studies were approved by the Animal Research Ethics Committee of UCD and the Health Products Regulatory Authority of Ireland.

## CRediT authorship contribution statement

**Ariyawan Tantipongpiradet:** Data curation, Formal analysis, Investigation, Methodology, Visualization, Writing – original draft, Writing – review & editing. **Reeta Davis:** Investigation, Methodology, Writing – original draft, Writing – review & editing. **Enrico A. Altieri:** Conceptualization, Funding acquisition, Writing – review & editing. **Kevin E. O'Connor:** Conceptualization, Formal analysis, Funding acquisition, Supervision, Writing – original draft, Writing – review & editing. **Keith J. Murphy:** Conceptualization, Data curation, Formal analysis, Funding acquisition, Investigation, Methodology, Project administration, Supervision, Visualization, Writing – original draft, Writing – review & editing.

## Declaration of competing interest

KJM and AT have no conflict of interest with respect to the research featured in this paper. RD is employed by and is a shareholder in Nova Mentis. KEOC and EAA are founders of and shareholders in Nova Mentis.

## Data Availability

Data will be made available on request.
