# Supplementary material for: Hydroxytyrosol confers resilience against the depressive, anxiogenic and cognition-disruptive effects of chronic stress
Source: Neurobiol Stress. 2026 May 22;42:100824. doi: 10.1016/j.ynstr.2026.100824 (PMC13235406; doi:10.1016/j.ynstr.2026.100824)
Supplement: Multimedia component 1 [file mmc1.pdf]

Supplemental Data

A

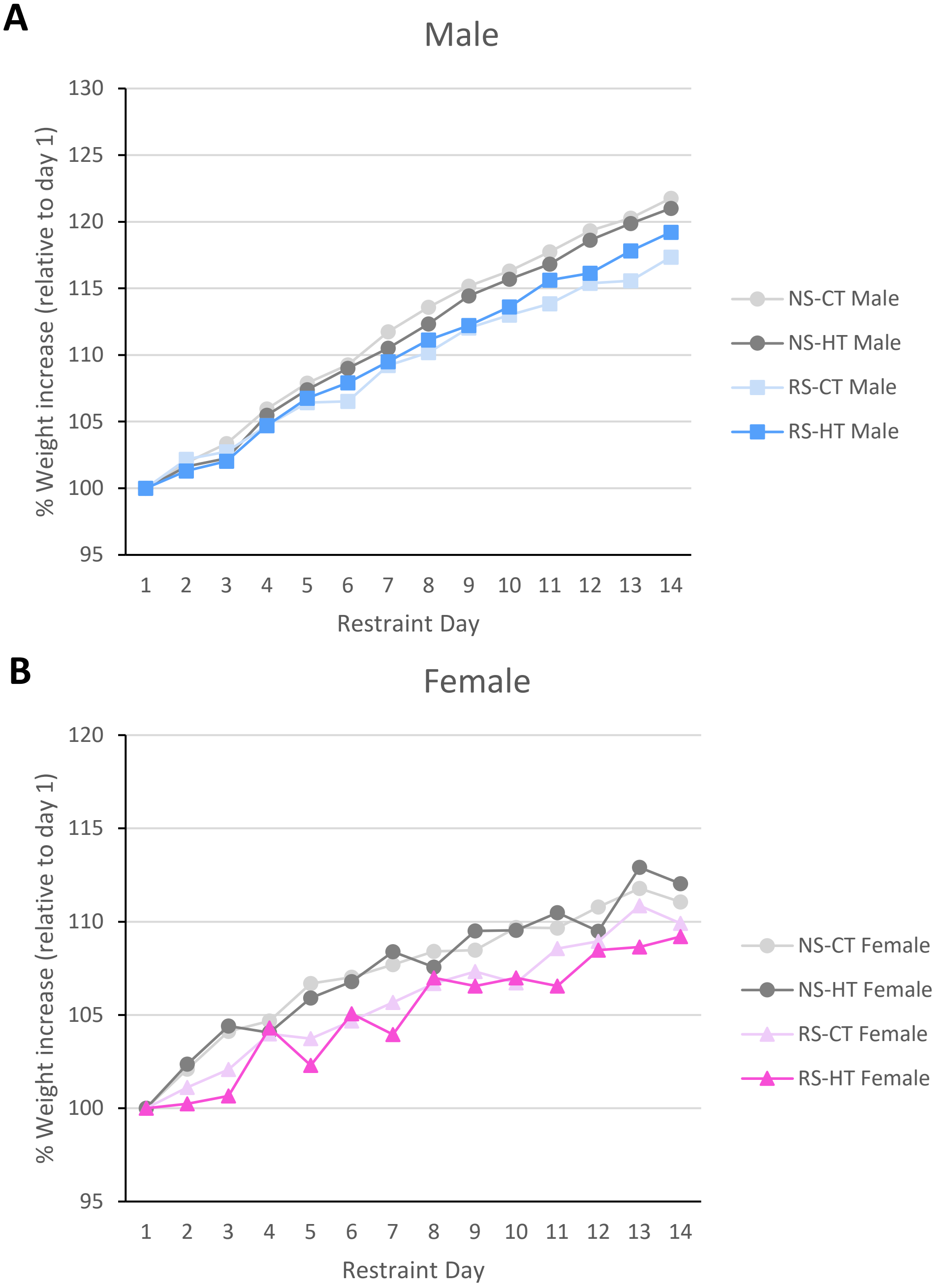

Figure S1: Effect of CURS and HT on weight gain. A: Body weight changes in male animals across the experimental period. CURS males exhibited lower weight gain compared to the control group. HT treatment demonstrated a trend towards resilience, supporting weight gain recovery. **B:** Body weight changes in female animals across the experimental period. Similar to males, CURS females showed reduced weight gain compared to the control level.

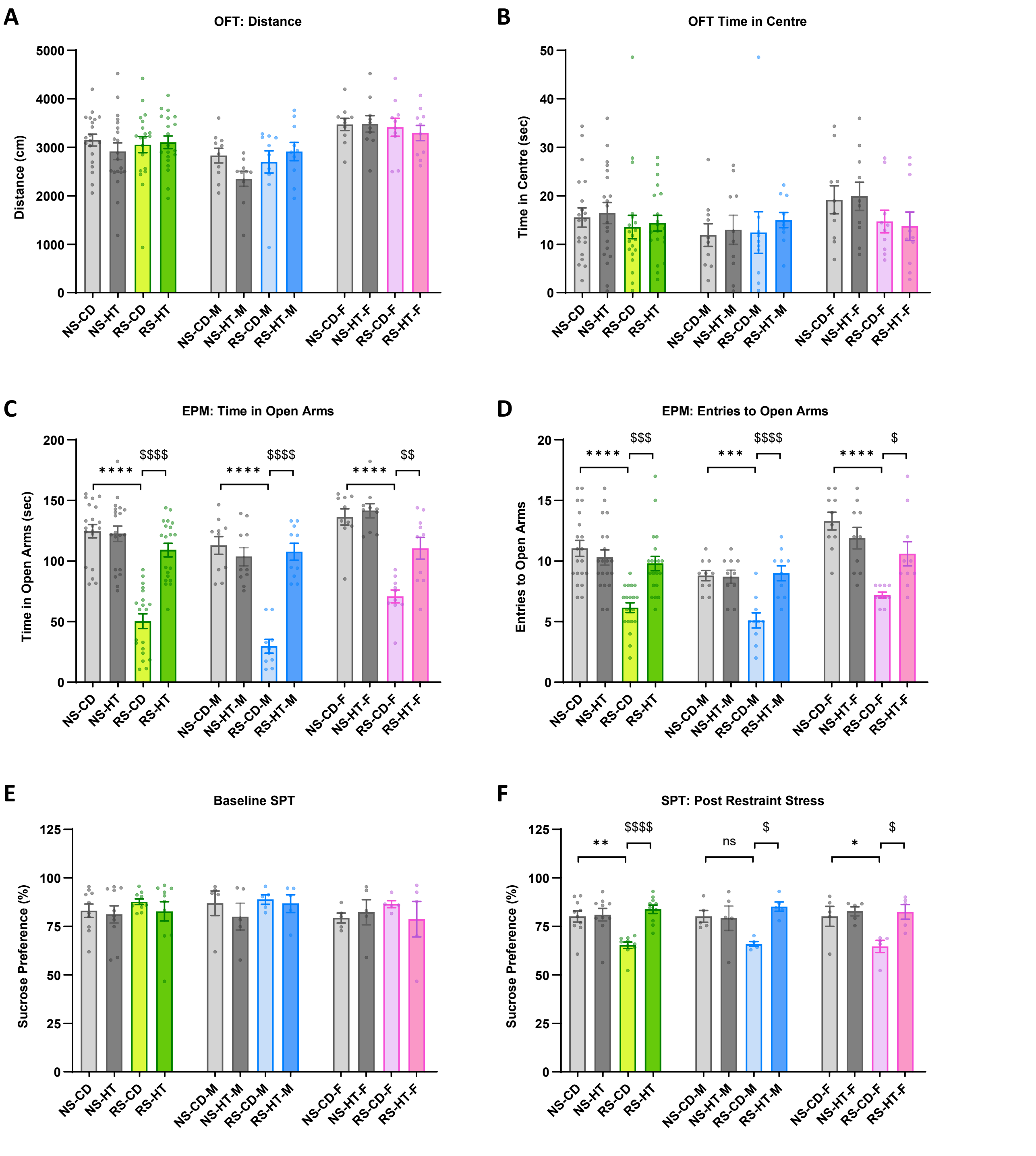

Figure S2: Effects of restraint stress and HT on locomotor activity, anxiety-like behaviour, and anhedonia. Open-field test showing total distance travelled (A) and time spent in the centre (B) during a 5-min session. CURS did not markedly alter locomotor activity but reduced centre exploration, indicative of heightened anxiety, whereas HT treatment partially normalized this behaviour. Elevated plus maze performance, represented by time spent in (C) and entries into (D) open arms. (E, F) Sucrose preference test assessing anhedonia before and after restraint stress, respectively. Baseline sucrose preference did not differ among groups. Following CURS, sucrose preference declined significantly, indicating reduced hedonic drive, while HT treatment preserved sucrose preference relative to CURS alone in both sexes. Data are mean ± SEM (n = 10 male (M) or female (F) animals per group; n = 20 when combined). Individual animals are represented by small circles. *P < 0.05, **P < 0.01, ***P < 0.001, ****P < 0.0001 vs. control (NS-CD); $ P < 0.05, $$ P < 0.01, $$$ P < 0.001, $$$$ P < 0.0001 vs. CURS animals (RS-CD) (two-way ANOVA with Tukey's post hoc test).

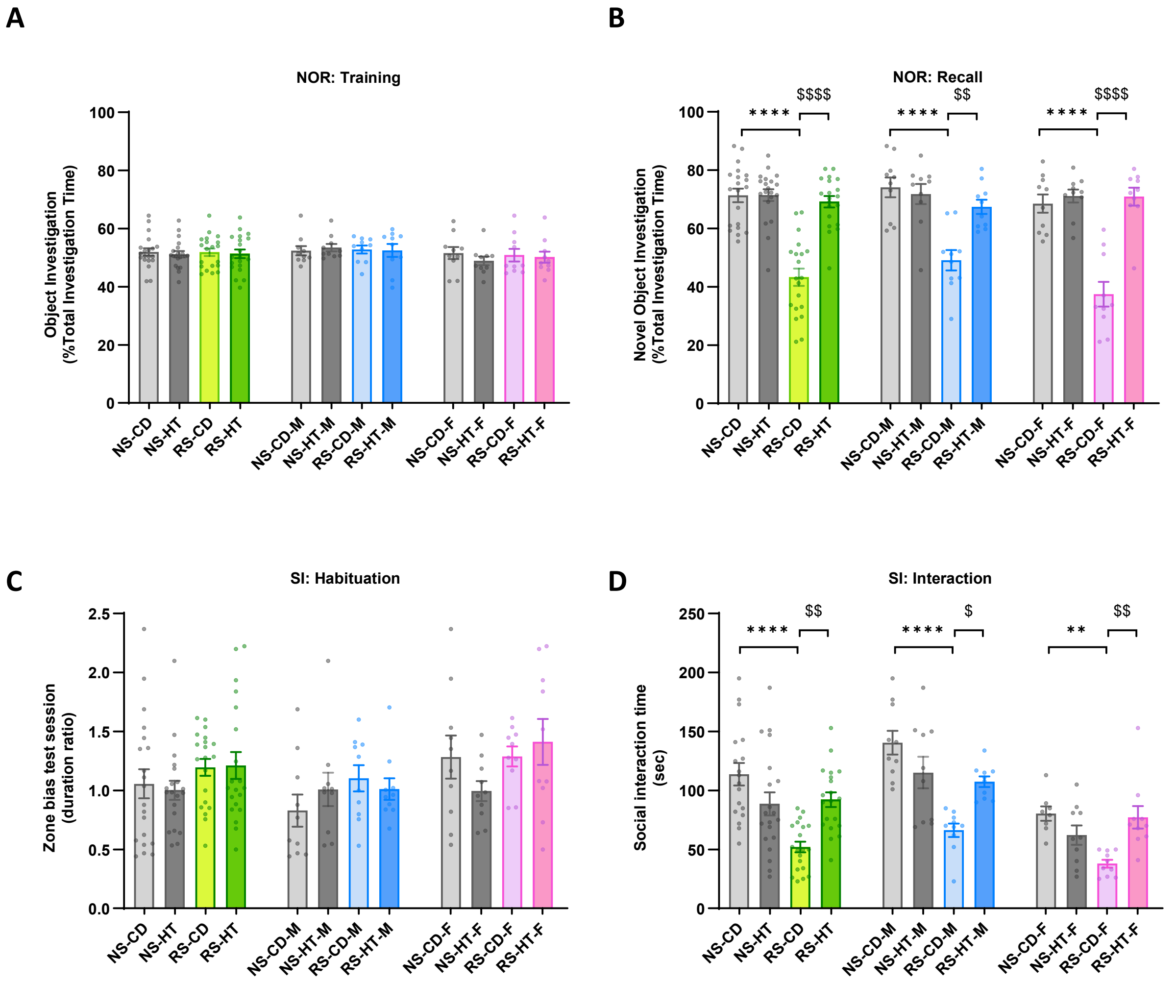

Figure S3: HT protects against stress-induced cognitive and social deficits. (A, B) Novel Object Recognition (NOR) test assessing working memory. Shown is the percentage of total investigation time spent on the novel object during the test phase. (C, D) Social Preference Activity Test assessing social behaviour. (C) Habituation phase; (D) total social interaction time. Data are mean ± SEM (n = 10 male (M) or female (F) animals per group; n = 20 when combined). Individual animals are represented by small circles. *P < 0.05, **P < 0.01, ***P < 0.001, ****P < 0.0001 vs. control (NS-CD); $ P < 0.05, $$ P < 0.01, $$$ P < 0.001, $$$$ P < 0.0001 vs. CURS animals (RS-CD) (two-way ANOVA with Tukey's post hoc test).

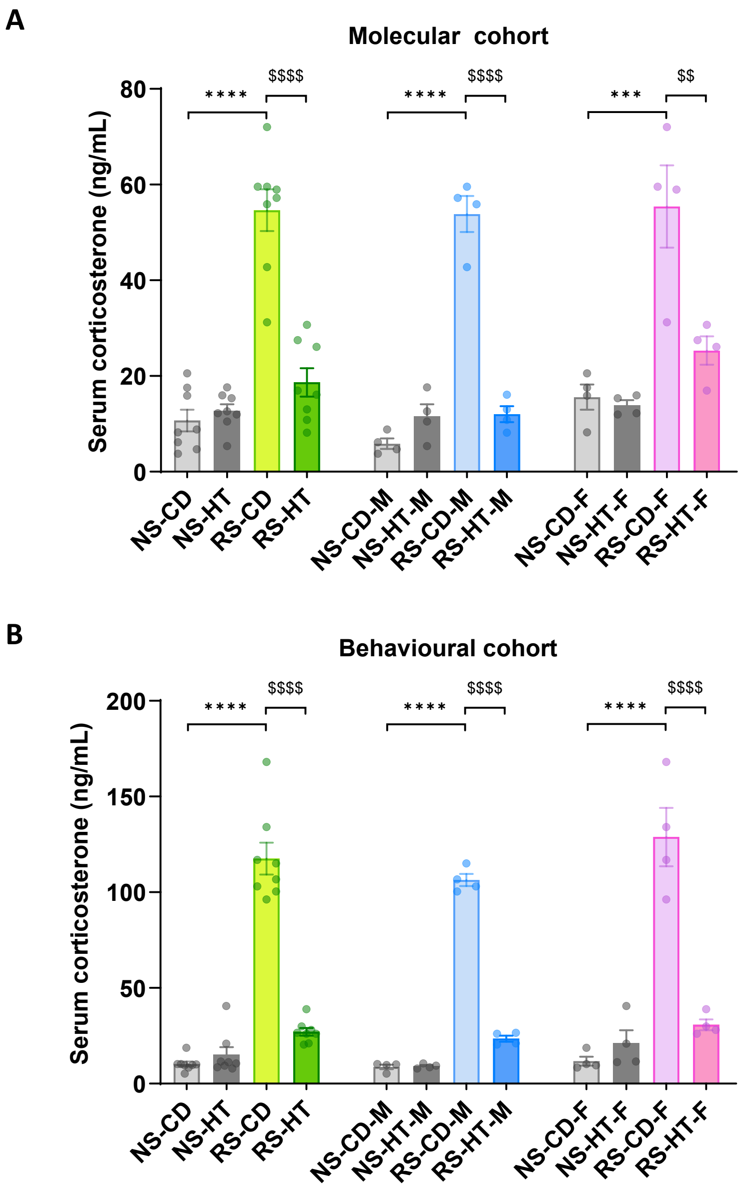

Figure S4: HT protects against stress-induced increase in serum corticosterone levels. HT treatment mitigates the elevation of serum corticosterone induced by chronic unpredictable restraint stress (CURS). **A:** Serum corticosterone levels in the Molecular cohort. CURS-treated animals exhibited significantly higher serum corticosterone levels compared to controls in the overall, male, and female groups, indicating increased stress reactivity. HT treatment significantly reduced serum corticosterone levels in the overall, male, and female groups, suggesting a strong protective effect of HT against chronic stress. **B:** Serum corticosterone levels in the Behavioural cohort. CURS-treated animals showed significantly elevated serum corticosterone levels compared to controls in the overall, female and male groups. HT treatment significantly reduced corticosterone levels in CURS-treated animals in the overall, female and male groups. Data are mean ± SEM (n = 4 male (M) or female (F) animals per group; n = 8 when combined). Individual animals are represented by small circles. *P < 0.05, **P < 0.01, ***P < 0.001, ****P < 0.0001 vs. control (NS-CD); $ P < 0.05, $$ P < 0.01, $$$ P < 0.001, $$$$ P < 0.0001 vs. CURS animals (RS-CD) (two-way ANOVA with Tukey's post hoc test).

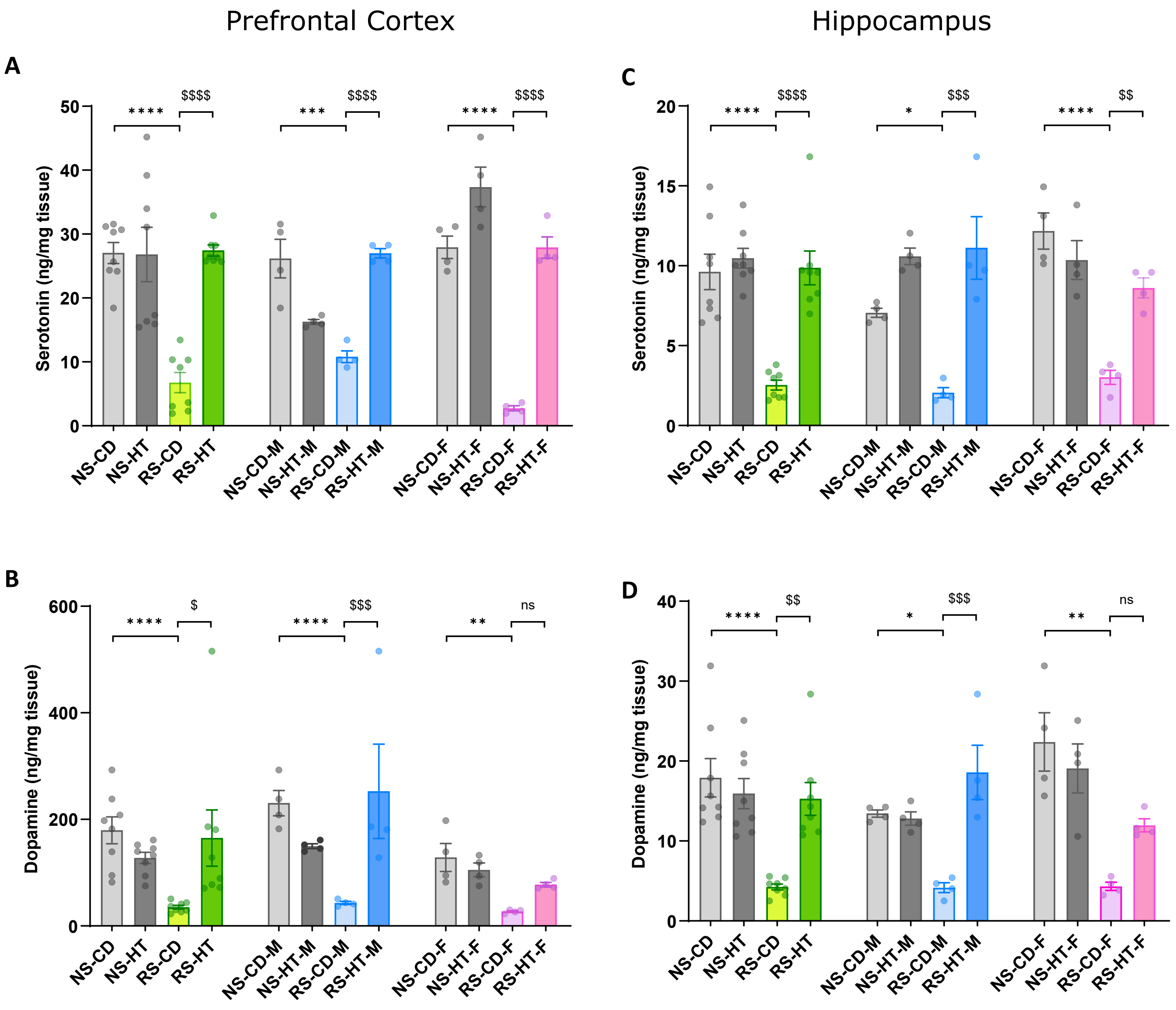

Figure S5. HT prevents stress-induced decreases in serotonin and dopamine in the prefrontal cortex and hippocampus. HT treatment reversed CURS-induced reductions in serotonin and dopamine levels across brain regions. (A) Prefrontal cortex serotonin: CURS significantly reduced serotonin in all groups, which was prevented by HT. (B) Prefrontal cortex dopamine: CURS reduced dopamine overall and in male and female groups. HT increased levels in overall and male groups, with no significant effect in females (ns). (C) Hippocampal serotonin: CURS decreased serotonin overall and in male and female groups. HT restored levels in all groups. (D) Hippocampal dopamine: CURS decreased dopamine in all groups. HT increased levels in overall and male groups, but not in females (ns). Data are mean ± SEM (n = 4 male (M) or female (F) animals per group; n = 8 when combined). Individual animals are represented by small circles. *P < 0.05, **P < 0.01, ***P < 0.001, ****P < 0.0001 vs. control (NS-CD); $ P < 0.05, $$ P < 0.01, $$$ P < 0.001, $$$$ P < 0.0001 vs. CURS animals (RS-CD) (two-way ANOVA with Tukey's post hoc test).

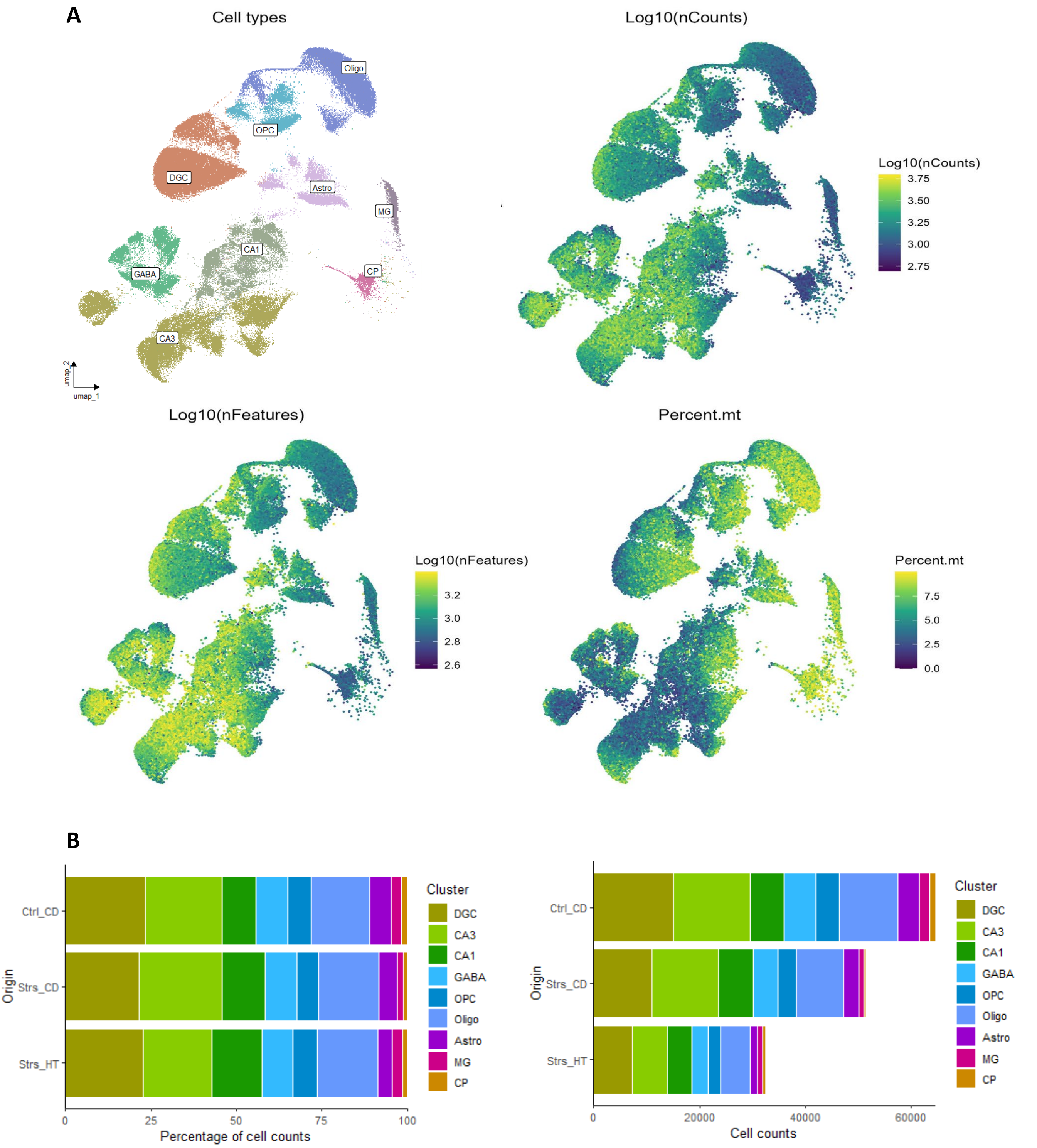

Figure S6: QC for single cell RNAseq transcriptional analysis of effect of stress and HT in hippocampus. A: UMAPs showing the cell type clusters, UMIs per cell (nCounts), genes per cell (nFeatures), and percentage of mitochondrial genes (percent.mt). B: Stacked bar graphs showing the percentage and number of each cell type for each group.

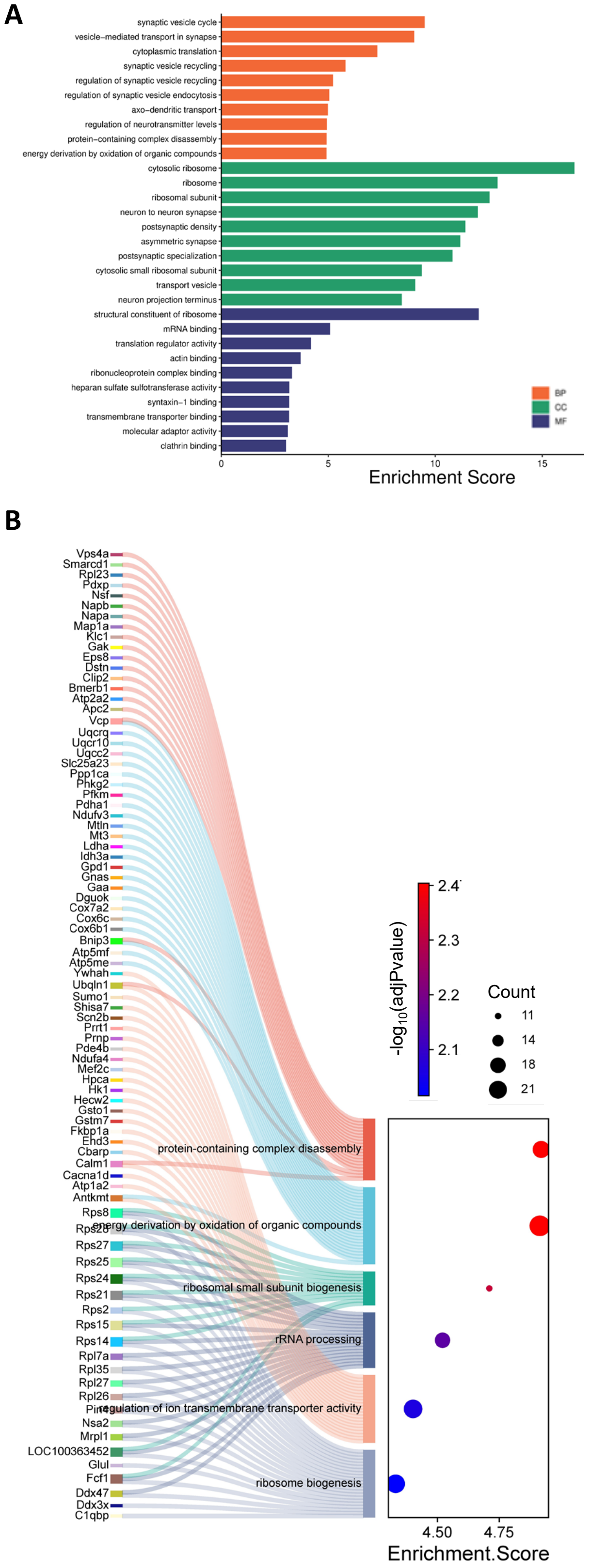

Figure S7: Single cell RNAseq transcriptional analysis of effect of stress. A: GO pathway enrichment analysis of stress DEGs. **B:** Sankey & dot plots showing DEGs among the top 30 most significant stress-associated GO Biological Functions related to protein synthesis, ion transport and cellular energetics. DEG: adj_p_value < 0.05 and $|\log_2 FC| \geq 0.3$.

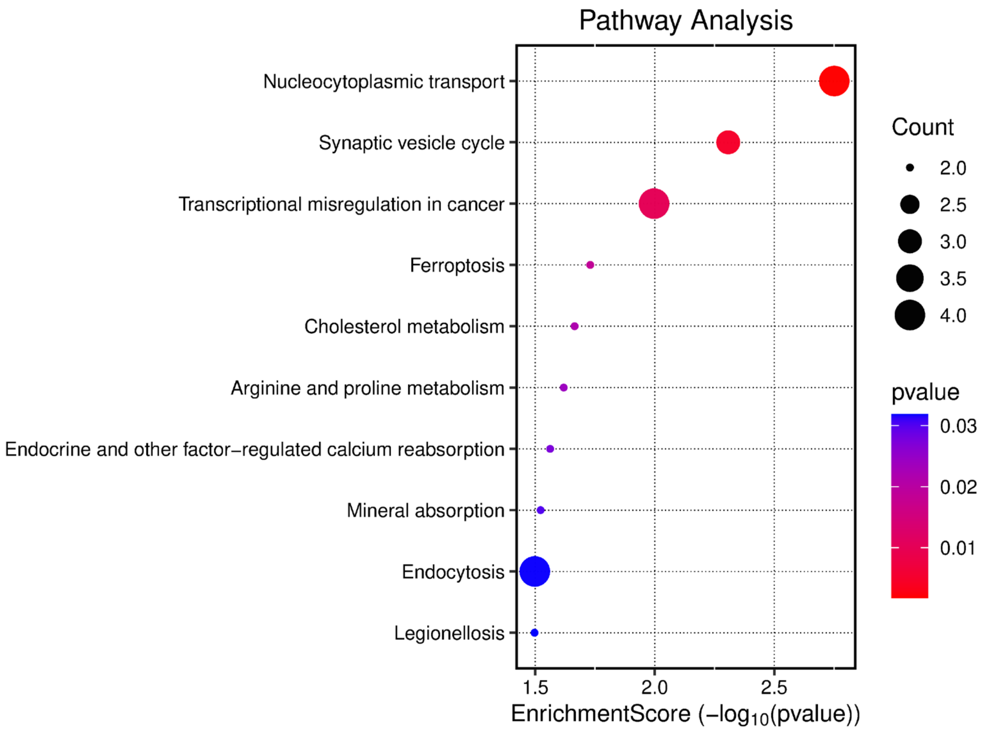

Figure S8: Single cell RNAseq transcriptional analysis of effect of HT on stress-associated dysregulation. Top 10 most significant KEGG pathways regulated by HT in CURS animals.

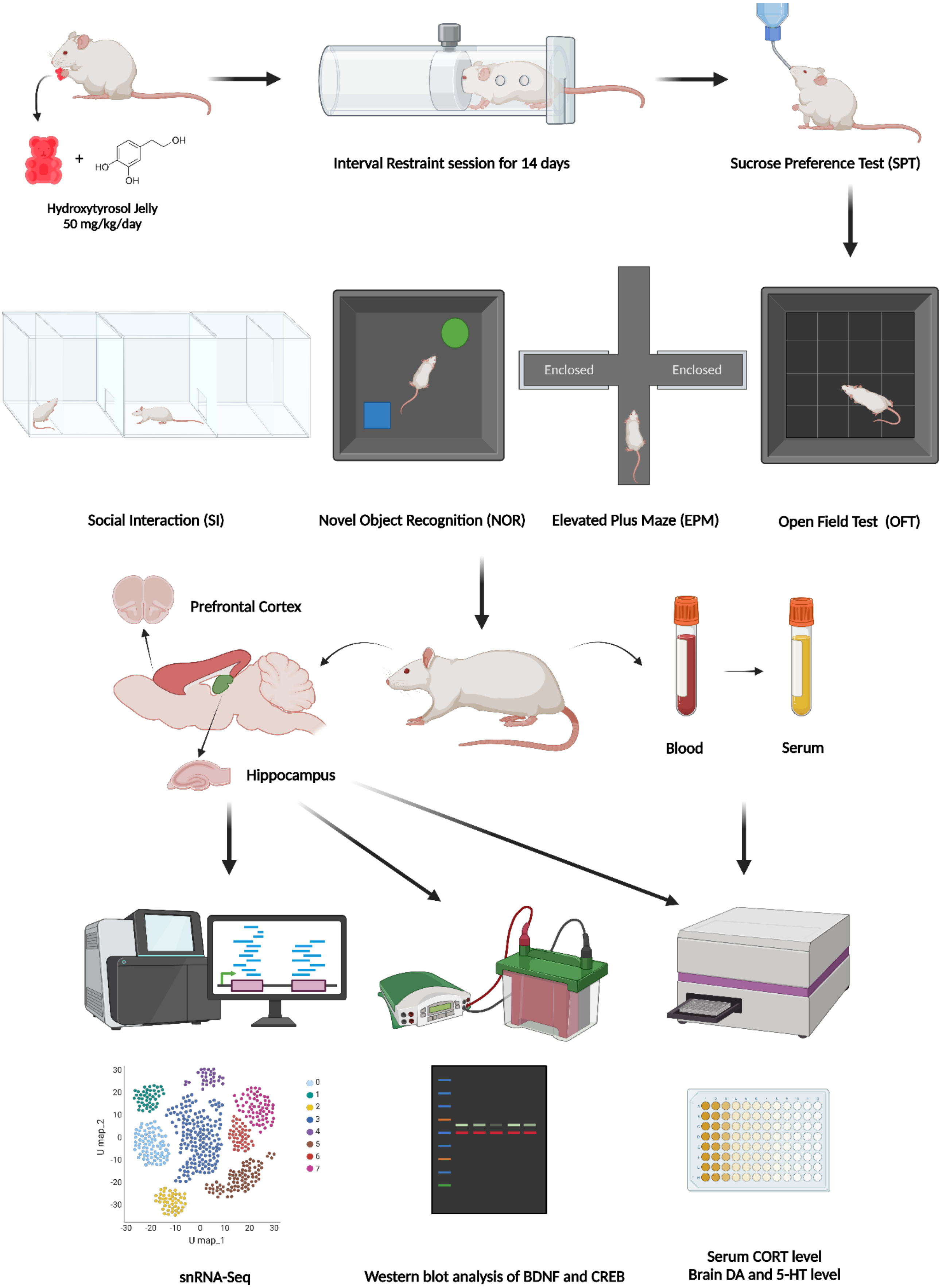

Graphical Abstract
